# UV-CD12: synchrotron radiation circular dichroism beamline at ANKA

**DOI:** 10.1107/S1600577515004476

**Published:** 2015-04-11

**Authors:** Jochen Bürck, Siegmar Roth, Dirk Windisch, Parvesh Wadhwani, David Moss, Anne S. Ulrich

**Affiliations:** a Institute for Biological Interfaces (IBG-2), Karlsruhe Institute of Technology (KIT), POB 3640, D-76021 Karlsruhe, Germany; b ANKA Synchrotron Radiation Facility, Karlsruhe Institute of Technology (KIT), POB 3640, D-76021 Karlsruhe, Germany; c Institute of Organic Chemistry, Karlsruhe Institute of Technology (KIT), Fritz-Haber-Weg 6, D-76131 Karlsruhe, Germany

**Keywords:** synchrotron radiation circular dichroism (SRCD), vacuum-UV, secondary structure, proteins, alignment of membrane-active peptides, oriented CD (OCD) of anisotropic samples

## Abstract

Synchrotron radiation circular dichroism (SRCD) is a rapidly growing technique for structure analysis of proteins and other chiral biomaterials. UV-CD12 is a high-flux SRCD beamline installed at the ANKA synchrotron, to which it had been transferred after the closure of the SRS Daresbury. The beamline covers an extended vacuum-UV to near-UV spectral range and has been open for users since October 2011. The current end-station allows for temperature-controlled steady-state SRCD spectroscopy, including routine automated thermal scans of microlitre volumes of water-soluble proteins down to 170 nm. It offers an excellent signal-to-noise ratio over the whole accessible spectral range. The technique of oriented circular dichroism (OCD) was recently implemented for determining the membrane alignment of α-helical peptides and proteins in macroscopically oriented lipid bilayers as mimics of cellular membranes. It offers improved spectral quality <200 nm compared with an OCD setup adapted to a bench-top instrument, and accelerated data collection by a factor of ∼3. In addition, it permits investigations of low hydrated protein films down to 130 nm using a rotatable sample cell that avoids linear dichroism artifacts.

## Abstract 2

UV-CD12 at ANKA and its current end-station are described, with a standard module for vacuum-UV synchrotron radiation circular dichroism of bio-macromolecules in the liquid state, and a unique module for macroscopically oriented lipid membranes (oriented circular dichroism).

## 1. Introduction   

Circular dichroism (CD) is defined as the differential absorption of left- and right-handed circular polarized light by chiral molecules. It is a well established spectroscopic technique in structural biology that complements high-resolution techniques such as X-ray crystallography or nuclear magnetic resonance (NMR). Although CD is a low resolution method it can provide structural properties of proteins and other chiral bio-macromolecules in their native environment (aqueous or membrane-bound). Numerous reports on the secondary structure composition and tertiary structure fingerprints of proteins have been published and the fundamentals of CD and examples of applications can be found in various reviews and textbooks (Fasman, 1996 ▶; Greenfield, 1999 ▶; Berova *et al.*, 2000 ▶, 2012 ▶; Kelly *et al.*, 2005 ▶).

In the last 15 years, synchrotron radiation circular dichroism (SRCD) has become an attractive extension beyond bench-top instruments for refined structure analysis of bio-macromolecules (Wallace & Janes, 2010 ▶; Hoffmann *et al.*, 2007 ▶; Jávorfi *et al.*, 2010 ▶; Hussain *et al.*, 2012*a* ▶,*b* ▶; Réfrégiers *et al.*, 2012 ▶). Compared with bench-top CD, SRCD provides about three orders of magnitude more intense photon flux below 200 nm. It thus offers a far better signal-to-noise ratio and extends the usable wavelength range down to ∼170 nm for aqueous protein solutions. Due to these advantages, only small amounts of precious biological samples are necessary, and the obtained spectra contain additional structural information. This allows for discrimination of more secondary structure elements with higher accuracy, *e.g.* for proteins with predominantly β-sheet or polyproline type-II conformation, because these spectra have strong peaks at low wavelengths (Nesgaard *et al.*, 2008 ▶; Brahms *et al.*, 1977 ▶) that are better distinguished from those of helical structures, *e.g.* due to their opposite sign in the wavelength range from 170 to 190 nm. Furthermore, high-quality measurements of proteins in the presence of strongly absorbing matrices, *e.g.* salts, buffers and detergent micelles, or highly scattering lipid vesicles are possible. This feature of SRCD offers access to structural information on systems such as membrane-bound proteins, fibril forming or aggregating proteins, or intrinsically disordered proteins that are difficult to analyze by high-resolution methods (Wallace & Janes, 2010 ▶; Hoffmann *et al.*, 2007 ▶; Jávorfi *et al.*, 2010 ▶; Hussain *et al.*, 2012*a* ▶,*b* ▶; Réfrégiers *et al.*, 2012 ▶). Therefore, SRCD is often combined very favourably with methods such as NMR or protein X-ray crystallography for characterizing the same protein sample, as a comprehensive picture can be obtained for correlating the structural properties of a biopolymer with its function (Wallace & Janes, 2010 ▶; Hoffmann *et al.*, 2007 ▶; Jávorfi *et al.*, 2010 ▶; Hussain *et al.*, 2012*a* ▶,*b* ▶; Réfrégiers *et al.*, 2012 ▶).

From the mid-2000s there has been a rapid growth in the number of new SRCD beamlines built, so currently a total of nine are operational and a few others are in the planning phase (see Table S2 in the supporting information). CD12 at the SRS (Daresbury Laboratory, UK) was a relatively new beamline (Clarke & Jones, 2004 ▶), producing consistently excellent scientific output over a five-year period. It was highly desirable to extend the working life of this beamline beyond the closure of SRS by transferring to another synchrotron facility. ANKA (Karlsruhe, Germany) and SRS are quite similar synchrotron facilities, so that a beamline that was originally designed for use at SRS was well suited for operation at ANKA. The bending-magnet radius and operating current of the two machines are identical, and the storage ring circumference is similar. The only significantly different parameter that had to be considered was the electron beam energy, which is 2.5 GeV at ANKA *versus* 2 GeV at SRS. Therefore, following the closure of the SRS, CD12 was transferred to Karlsruhe in 2009, and adapted to the vacant port B2.2.5 on the north side of the ANKA storage ring. After comprehensive commissioning work the beamline became active again in autumn 2011 for the user community under the new name UV-CD12. The beamline serves the German as well as the international community of structural biologists, biophysicists and chemists.

## 2. Beamline overview   

### 2.1. Installation of UV-CD12 at ANKA   

**Figure 1:**
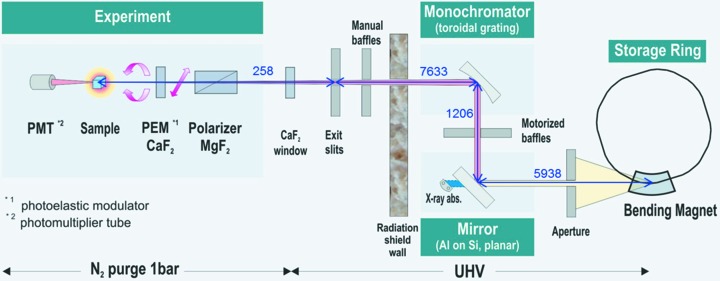
Schematic diagram of the UV-CD12 beamline at ANKA, showing the layout of the main optical components (not drawn to scale). The numbers refer to the relative distances of the main optical elements in millimetres.

The excellent performance and reliability of CD12 exhibited during the five-year period of user operation at the SRS was largely due to its well-conceived and simple optical design, which has been described in detail earlier (Clarke & Jones, 2004 ▶). Therefore, the chief design goal for installing the beamline at ANKA was to maintain the same optical scheme shown in Fig. 1 ▶ with as little modification as possible. The Daresbury layout of CD12 could not be exactly duplicated at ANKA, because the storage ring radiation shield wall has no roof. However, a near duplication was achieved by shortening the vertical arm after, and a corresponding lengthening of the horizontal arm of the optical path before, the plane extraction mirror, to maintain the 7.144 m distance from the source point to the grating. The 45% increased optical path from source point to extraction mirror compensates for the additional heat load caused by ANKA’s higher electron beam energy. The grating-to-sample distance of 7.9 m remains unchanged. These modifications in beamline design required an elevation of the floor for the experimental area inside the hutch to a level of 1.61 m above the ground.

The main components of the beamline are the front-end, the primary mirror chamber, the monochromator chamber with motorized beam-defining baffles, and the post-mono segments containing manual baffles, as well as the acoustic delay and exit slit sections (see supporting information Fig. S1 for a three-dimensional view of UV-CD12). The front-end is completely new and consists of a water-cooled beam absorber, a gate valve and a fast closing shutter. Two additional vacuum valves, which are located between primary mirror and monochromator chamber and before the exit slit section, allow the different compartments to be separated from each other and from the storage ring.

### 2.2. Optical layout of the beamline and experimental setup   

CD12 at Daresbury extracted 35 mrad of the beam in the horizontal and 7 mrad in the vertical plane. For approaching these values, the original dipole chamber of the bending-magnet at port B2.2.5 at ANKA was replaced by a new one with a doubled acceptance angle of 30.8 mrad in the horizontal and 3.7 mrad in the vertical, resulting in a beam size of 183 × 22 mm at the water-cooled mirror that absorbs the X-ray component of the synchrotron radiation. The planar mirror deflects the polychromatic UV/VIS beam upwards at 90° towards a toroidal holographic diffraction grating that re-directs horizontally the monochromatic beam through the acoustic delay tube to the sample. The grating roll and yaw mechanics inside the monochromator chamber have been motorized. It was important to allow for remote adjustment, because the monochromator, in contrast to the Daresbury setup, is located behind the radiation shield wall at ANKA and is not accessible during beamline operation. Due to the toroidal shape of the grating, the beam is focused in the vertical plane on the exit slit and in the horizontal direction on the sample position. The optical components and key parameters of the beamline are specified in Table 1 ▶.

Pre- and post-monochromator baffles, each consisting of four independently adjustable blades, are implemented to select any part of the beam to control beam polarization characteristics and diminish stray light. The focused UV synchrotron beam leaves the ultra-high vacuum (UHV) *via* a CaF_2_ exit window and enters into the nitrogen-purged regime of the experimental setup, which is mounted on an optical bench placed behind the exit window. Fast sample changing at atmospheric pressure is enabled by the N_2_ environment, which is still sufficiently transparent for SRCD measurements down to ∼130 nm.

**Figure 2:**
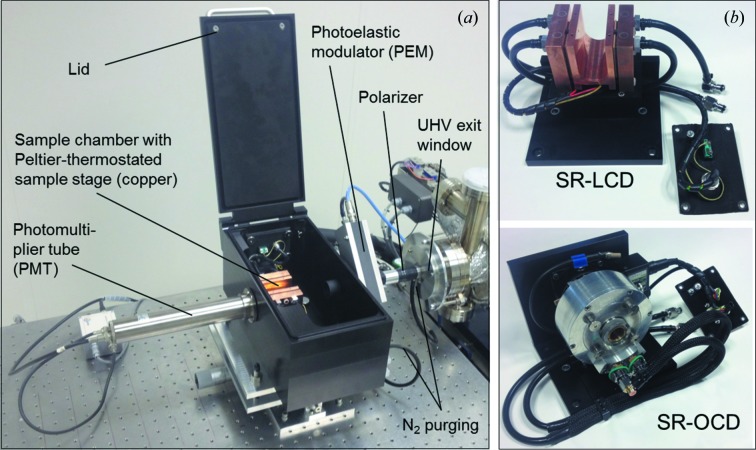
(*a*) Photograph of the experimental end-station for steady-state SRCD measurements, with the lid of the sample chamber opened. (*b*) Modules for liquid-state SRCD with Peltier elements for thermostating/thermal scans in the temperature range from 275 to 368 K, and SR-OCD (for oriented samples) with a water-thermostated OCD cell mounted on a rotation stage; these modules can be mounted alternately in the sample chamber.

A photograph of the end-station for steady-state SRCD measurements is shown in Fig. 2 ▶. The synchrotron beam is intrinsically linearly polarized in the horizontal plane. For maximizing the degree of linear polarization a Rochon polarizer is integrated in the setup. After that, the light is transmitted through a photo-elastic modulator (PEM) at 45° with respect to the Rochon axes converting the linear to left- and right-handed circular polarization with a modulation frequency of 50 kHz. A circular-shaped aperture behind the PEM serves as a beam stop for the extraordinary beam generated by the Rochon polarizer to prevent light with the wrong polarization from reaching the detector. Polarizer and PEM are mounted on the optical bench and integrated into light-tight nitrogen-purged chambers. The circularly polarized light transmitted through the sample mounted in the sample chamber is detected by a photomultiplier tube (PMT) with MgF_2_ end window. Alternatively, other detectors, *e.g.* a solar-blind PMT with a sensitivity restricted to the VUV to near-UV range, can be installed. The output signal of the PMT is processed by a preamplifier, and separated in its DC and AC components by a digital lock-in amplifier (Stanford Research Systems, SR 850). The AC signal is detected by the lock-in using the PEM modulation frequency as a reference. An in-house-developed servo-loop system regulates the high voltage (HV) applied on the PMT to retain a constant DC signal. From the ratio of the AC and DC components, multiplied by an instrument-dependent amplification factor, the final CD signal is calculated. The signal magnitude is calibrated using a camphor sulfonic acid (ammonium salt) standard solution. A typical SRCD calibration spectrum together with the specification data for wavelength calibration, spectral resolution and stray light are given in Figs. S2–S5 in the supporting information. It is advantageous to record the CD and the absorption spectrum of the chiral sample at the same time and with identical sample configuration. Therefore, the HV applied on the PMT (ET 9406B), the PMT gain and the beam current of sample and reference measurement are always used to determine the sample absorbance according to a procedure described by Sutherland (1996 ▶).

Two different modules for synchrotron radiation based liquid-state CD (SR-LCD) of protein solutions on the one hand, and for oriented CD (SR-OCD) of peptides embedded in macroscopically aligned lipid bilayers on the other hand, can be alternately mounted in the sample chamber [*cf*. Figs. 2(*a*) and 2(*b*) ▶]. This chamber is positioned between the PEM housing and the PMT, and is also purged with nitrogen at a flow rate of 20 L min^−1^.

The SR-LCD module consists of a U-shaped copper block for mounting cuvette holders with optical cells, and it is temperature-controlled by two Peltier heat pumps. The holders can accommodate different types of cells for aqueous protein solutions, *e.g.* cylindrical cells with optical path lengths of 50 µm to 5 mm (121.000-QS), or demountable cells with path lengths of 2–20 µm (124-QS, 124-CaF_2_ both from Hellma, Müllheim, Germany), in which the sample is deposited between two disk-shaped windows made of quartz glass or CaF_2_ (Wien & Wallace, 2005 ▶). Moreover, rectangular quartz glass cells used in bench-top CD with optical path lengths of up to 1 cm can be mounted in the U-shaped stage *via* an adapter made from copper.

The module for SR-OCD contains a cell which allows the membrane alignment of helical peptides and protein fragments in mechanically oriented lipid bilayers to be measured under controlled hydration and temp­erature conditions. The automated OCD cell has been developed and built in-house, as described in detail earlier (Bürck *et al.*, 2008 ▶). The optical path runs along the cylindrical axis of the cell, *i.e.* normal to the quartz glass window carrying the oriented samples. To avoid linear dichroism and birefringence artifacts the cell is mounted on a motor-driven computer-controlled rotation stage. Averaging of OCD spectra taken at eight different angles around the beam axis allows compensation for these artifacts. This rotatable cell is also suitable for measuring protein films under low hydration when deposited on a CaF_2_ window.

## 3. Ancillary facilities   

The control of the different optical, electric and thermoelectric modules of the experimental end-station, as well as spectral data acquisition and storage, is accomplished *via* in-house software developed under the LabView programming environment. This software also includes very flexible and user-friendly Macro-programming, which allows launching automated thermal scan or time series experiments. Post-processing of SRCD spectra can be performed on-site with the *CDtool* software package (Lees *et al.*, 2004 ▶, http://cdtools.cryst.bbk.ac.uk/).

A work-bench for basic biochemical sample preparation is available for users in the UV-CD12 experimental hutch, which includes standard laboratory consumables and the following equipment: degassing (vacuum chamber), vortexer/shaker, centrifuge, ultrasonic baths, NanoDrop 2000c UV spectrometer for protein or DNA concentration determination, a small fume hood and a fridge/freezer (253 K). In a neighbouring building just 150 m away from the beamline an ice machine, a 193 K freezer, a high-precision analytical micro-balance and pH meters are provided by the off-line CD laboratory of the IBG-2. For more elaborate work, *e.g.* complementary bench-top CD measurements, this laboratory is equipped with a Jasco J-815 spectropolarimeter as well as a Shimadzu UV 2100 absorption spectrometer. Moreover, for collaboration projects of external users with the beamline staff, full access to the structural biology laboratories of IBG-2 is offered, including facilities for peptide synthesis and purification, recombinant protein production, biological facilities certified for safety level S1, as well as comprehensive solid-state NMR structure analysis (*e.g.* using the same oriented samples as for OCD).

## 4. Facility access   

UV-CD12 is a so-called collaborative research group (CRG) beamline, operated by ANKA’s neighbouring institute IBG-2. Under this model the institute is responsible for operation of the beamline, and ANKA provides the necessary infrastructure services. 30% of beam time is available to German and international external users *via* ANKA’s standard peer reviewed application procedure (https://proposal.anka.kit.edu/anna/). There are two calls for proposals each year with deadlines for submission on 15 January and 30 June.

In exchange for the gift of the beamline, a 20% beam time contingent has been granted to the UK user community for a five-year period, without going through the peer review procedure [starting from the date when UV-CD12 was opened for users at ANKA; contract with the Science and Technology Facilities Council (STFC), Daresbury Laboratory, UK]. Nevertheless, each of these projects is evaluated retrospectively by the ANKA Peer Review Committee. The remaining 40% of the beam time (+10% maintenance) is used by IBG-2 for in-house research and to support other KIT internal projects within the ‘BioInterfaces’ program of the Helmholtz Association.

## 5. Highlights   

**Figure 3:**
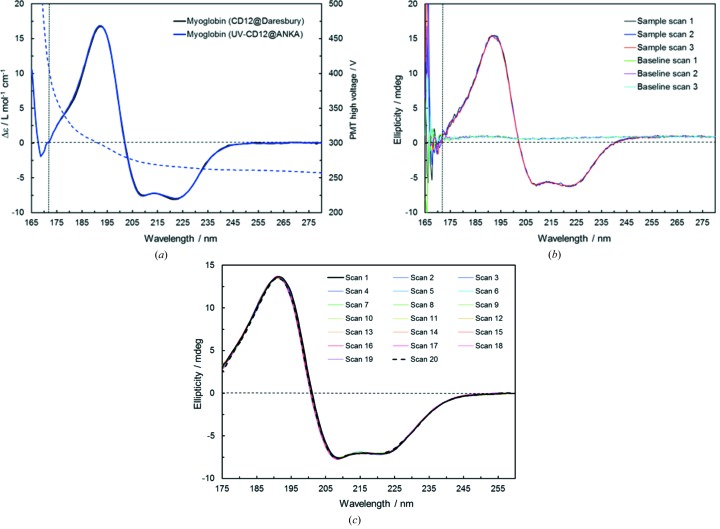
(*a*) Comparison of SRCD spectra of myoglobin in aqueous solution, recorded on UV-CD12 at ANKA and on CD12 at Daresbury [spectrum was taken from the protein circular dichroism data bank: PCDDBID CD0000047000 (Lees *et al.*, 2006 ▶)]; the dashed curve shows the HV applied on the PMT (ET 9406B) during the measurement at UV-CD12 and the vertical dotted line indicates the cut-off wavelength, which is typically reached at a HV of ∼400 V; data above this cut-off HV are not reliable. (*b*) Raw data of the myoglobin sample and H_2_O baseline spectra collected on UV-CD12; a 300 ms lock-in amplifier time constant and a 1.5 s dwell time have been used; the spectrum presented in (*a*) was averaged over the three raw spectra and the average of the three baseline spectra was subtracted and after Savitzky-Golay smoothening the ellipticities were converted to Δ∊ using the mean residue molar concentration of the protein and the cuvette path length. (*c*) 20 consecutive scans of human serum albumin, a protein that is highly sensitive to UV-radiation-induced denaturation; the first and last scan are black solid and dashed lines, respectively; no decrease of the signal magnitude is observed for all spectra, which proves the absence of any denaturation effects even at the conditions close to the maximum electron beam current of 160 mA used for this experiment.

In Fig. 3(*a*) ▶ the aqueous solution spectra of myoglobin are compared that were measured at UV-CD12 (with the new SR-LCD setup) and at CD12 in Daresbury (with the old setup). The two spectra are virtually superimposable and demonstrate that the same high performance level is obtained with the beamline installed at ANKA. It provides reliable measurements in aqueous solutions down to ∼170 nm, although the photon flux has been deliberately limited to 1 × 10^12^ photons s^−1^ (*cf*. Table 1 ▶) by adjusting the baffles to a more closed position to avoid UV-induced protein denaturation, which had been frequently encountered under the Daresbury photon flux conditions (Miles *et al.*, 2008 ▶). Nevertheless, an excellent signal-to-noise ratio can be obtained under these conditions as seen from the raw data of the myoglobin sample scans and the corresponding baseline scans presented in Fig. 3(*b*) ▶. The low root-mean-squared noise level at different wavelengths is specified in Table S1 in the supporting information. Moreover, due to the reduction of the photon flux by a factor of ∼15 compared with the SRS photon flux conditions, no UV-radiation-induced protein denaturation is observed at UV-CD12. This is clearly demonstrated in Fig. 3(*c*) ▶ for the protein human serum albumin (HSA) which is known to be highly sensitive to UV denaturation effects (Miles *et al.*, 2008 ▶) and does not show any spectral decrease in 20 consecutive scans even under the maximum photon flux conditions applied here. Denaturation of HSA can only be induced if the sample is exposed to the SRCD beam at 170 nm for an extended time of 10 min at each consecutive scan (see Fig. S6). However, such a measurement condition is never applied in typical user experiments, where the monochromator after the scan moves immediately back to the start wavelength, *i.e.* to a long-wavelength position where the light has much lower energy and waits here before a new scan starts. The maximum photon flux density of 0.8 × 10^11^ photons s^−1^ mm^−2^ at UV-CD12, which can be calculated from the photon flux and the beam size given in Table 1 ▶, is quite close to the flux density threshold value for denaturation, which was indicated as approximately 0.4 × 10^11^ photons s^−1^ mm^−2^ by Miles *et al.* (2008 ▶). The photon flux density at UV-CD12 is thus a compromise between reaching a good signal-to-noise ratio and avoiding protein denaturation, and a further increase in photon flux density would not bring any advantage for SRCD measurements.

**Figure 4:**
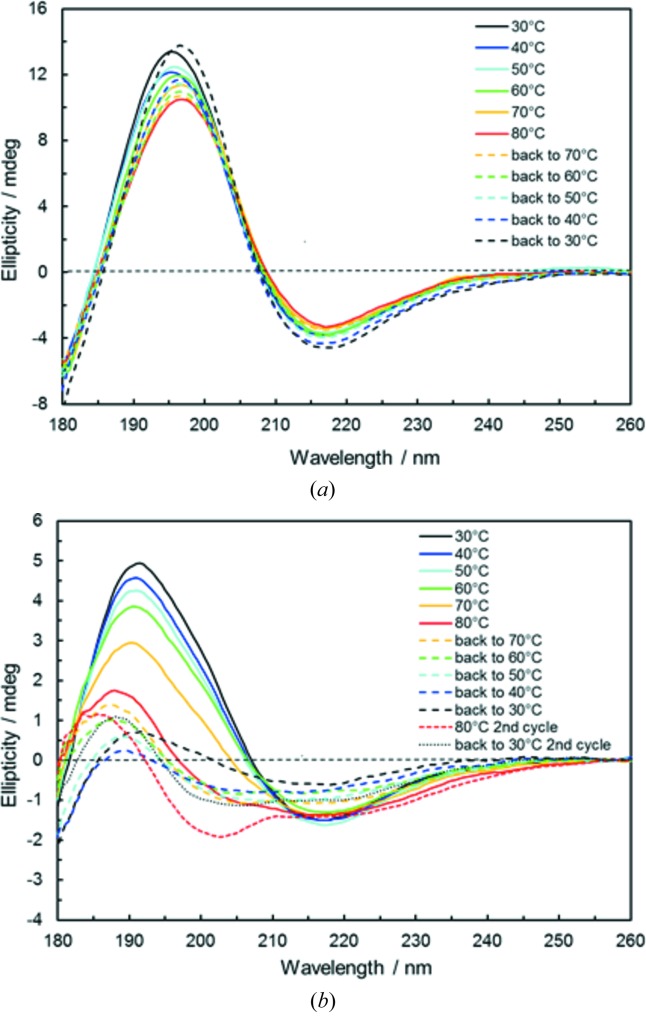
Thermal denaturation studies of the model antimicrobial peptide (KIGAKI)_3_. (*a*) The wild-type peptide forms β-pleated aggregates in DMPC/DMPG 3:1 liposomes (P/L 1:50), as can be deduced from the characteristic spectral line shape of the SRCD spectra. Upon heating up to 353 K, only a slight and reversible decrease in β-sheet fraction is observed. (*b*) For a mutant peptide, in which Ile-8 was replaced by a rigid fluorinated d-amino acid, extensive and irreversible unfolding of the β-pleated aggregates is observed, and even the formation of small helical fractions can be stated for the same heating/cooling cycle.

Another example of high-quality SRCD spectra recorded at UV-CD12 down to 180 nm in the presence of strongly scattering lipid vesicles at elevated temperatures is given in Fig. 4 ▶. (KIGAKI)_3_ is an amphiphilic antimicrobial peptide that lyses bacterial cell membranes and forms amyloid-like β-pleated fibrils in a lipidic environment (Wadhwani *et al.*, 2012 ▶, and references therein). Panels (*a*) and (*b*) show SRCD spectra of the wild-type peptide and a mutant, respectively, in DMPC/DMPG 3:1 liposomes obtained during a thermal scan from 303 to 353 K and cooling back to 303 K. By replacing Ile-8 with a sterically demanding fluorinated d-amino acid, the robust β-pleated amyloid-like structure of the wild-type peptide can be considerably destabilized, and the β-sheet aggregates of the mutant remain irreversibly disrupted upon heating up the sample. The incorporation of a single rigid d-amino acid into this fibril-forming peptide nicely demonstrates a simple strategy to prevent peptide aggregation, which often influences the biological activities of designer peptides and may lead to malfunction and disease (Wadhwani *et al.*, 2013 ▶).

**Figure 5:**
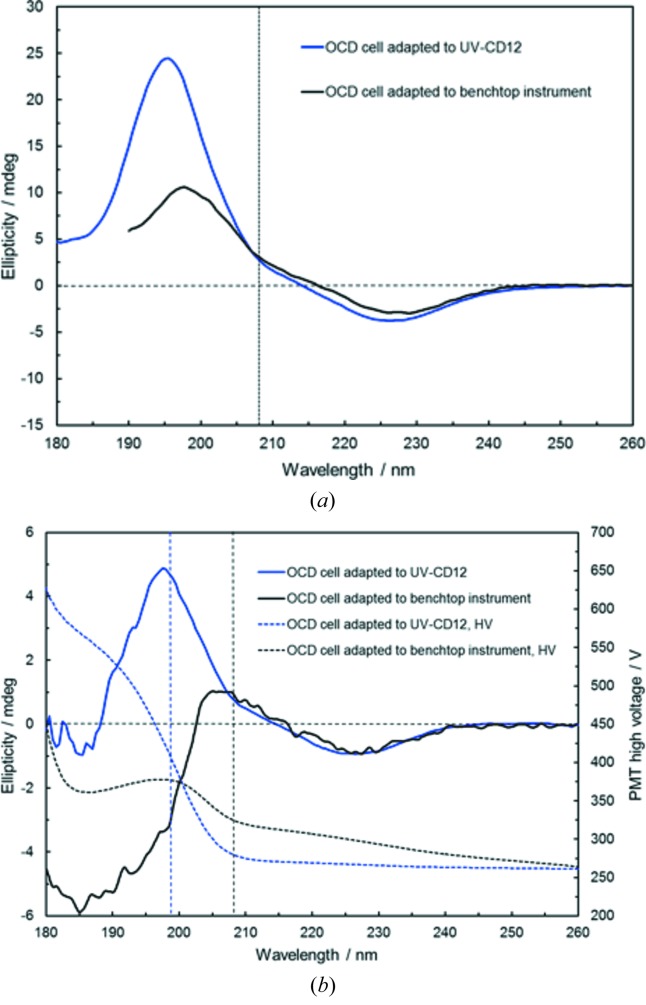
(*a*) Comparison of synchrotron radiation based and bench-top instrument based OCD spectra of the same sample (transmembrane segment of PDGFRβ) in fully hydrated oriented DEiPC lipid bilayers (P/L 1:50). The spectra exhibit the typical line shape of a helical fold, and an inserted transmembrane alignment of the peptide in the lipid bilayer can be clearly deduced from the absence of the negative band at 208 nm (see dashed vertical line), given the superior quality of the synchrotron radiation based OCD spectrum. (*b*) A second comparison of UV-CD12 and J-810 based OCD spectra of an identical sample: here PDGFRβ in fully hydrated DEiPC lipid bilayers has been measured at a P/L of 1:500, *i.e.* close to the detection limit; here the better signal-to-noise ratio of the SRCD based data is obvious and the SRCD data are reliable down to 198 nm, while the spectrum collected at the bench-top instrument due to the extreme background absorption of the unsaturated lipid and the low photon flux reaching the PMT detector exhibits undependable signals ≤208 nm and a strong kink with negative ellipticities at wavelengths <200 nm.

In the next example shown in Fig. 5(*a*) ▶, we directly compare OCD spectra of the very same sample measured in the same cell that was adapted either to the SR-OCD module or to the OCD setup on a model J-810 Jasco bench-top spectrometer. Both spectra show the hydrophobic transmembrane segment from the platelet-derived growth factor receptor beta (PDGFRβ), an integral membrane protein that was reconstituted in macroscopically oriented DEiPC lipid bilayers (see the supporting information). From the typical line shape in OCD (and of course by liquid-state CD of the peptide in a vesicle sample) an α-helical conformation can be clearly confirmed. Moreover, a distinct transmembrane orientation can be deduced, given the complete absence of the negative ‘fingerprint’ band around 208 nm, whose intensity is a measure of the helix tilt angle (Muhle-Goll *et al.*, 2012 ▶; Bürck *et al.*, 2008 ▶). At wavelengths <200 nm a 2.5-fold more intense signal magnitude of the positive band around 196 nm has been found for the synchrotron radiation based data. This fact can be explained by the enhanced photon flux at UV-CD12 as compared with the J-810, which allows a constant exit slit width to be maintained over the entire spectral range and thus guarantees a genuinely fixed spectral bandwidth of 1 nm. OCD experiments using the J-810 bench-top CD spectrometer have to be performed in automated slit mode, which means that the slit is gradually opened up at lower wavelengths due to the poor photon flux. This opened-up slit typically leads to an increased spectral bandwidth and hence to a reduction in signal magnitude and spectral distortions of the corresponding CD band (*cf*. Fig. S7 where OCD spectra of PDGFRβ in oriented lipid bilayers have been measured at varying fixed slit width).

A second effect contributing to the enhanced magnitude of the OCD signal is the 4.3-fold larger beam size of the J-810 (4 × 13 mm) compared with UV-CD12 (2.5 × 4.8 mm, see Table 1 ▶). Inhomogeneities in the same rotated oriented lipid bilayer sample present on average more empty areas for the J-810 beam geometry compared with the UV-CD12 beam, which hits the sample in a smaller area around the centre representing a more homogeneous lipid bilayer zone than zones at the outer rim. In a control experiment we have measured a peptide sample using the OCD setup of the J-810 with a pinhole inserted in front of the OCD cell, which mimicked the UV-CD12 beam geometry, and without the pinhole, *i.e.* with the ‘normal’ beam size. The OCD spectra are shown in Fig. S8 and a 40% enhanced signal magnitude was observed with the inserted pinhole, although due to the reduced beam size the signal obtained with the J-810 was very noisy at wavelengths <200 nm. Thus, we can conclude that both the spectral bandwidth and the beam size effects will contribute to the enhancement in signal magnitude for experiments performed with the UV-CD12 OCD setup.

In Fig. 5(*b*) ▶ a second comparison of OCD spectra of an identical sample measured in exactly the same cell with both instruments is presented illustrating the enhanced UV penetration of the UV-CD12 setup for a highly absorbing peptide/lipid sample. Here, the PDGFRβ transmembrane segment has been measured in fully hydrated DEiPC lipid bilayers at a peptide-to-lipid (P/L) ratio of 1:500, *i.e.* close to the detection limit. This sample with a very low peptide concentration in a high excess of an unsaturated lipid clearly reveals the better signal-to-noise ratio of the SRCD based data, which are reliable down to 198 nm. The spectrum collected at the bench-top instrument due to the extreme background absorption of the DEiPC lipid exhibits much higher noise levels over the whole spectral range, and due to the low photon flux reaching the PMT detector the ellipticity signals ≤208 nm are not reliable resulting in a strong kink with negative ellipticities at wavelengths <200 nm.

**Figure 6:**
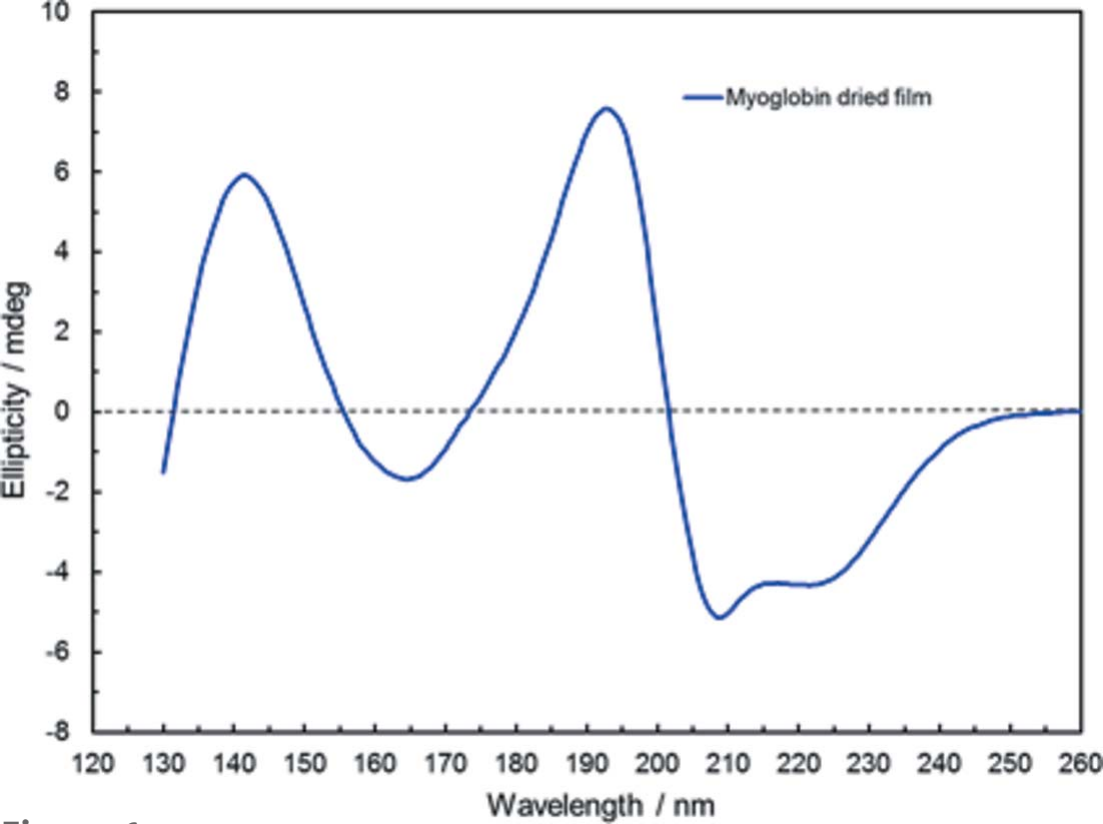
SRCD spectrum of a dried myoglobin protein film: here, due to the virtual absence of H_2_O background absorption, the wavelength range can be extended down to 130 nm.

An add-on feature of the rotation stage of the SR-OCD module is the possibility to measure protein films under low hydration, which have been deposited from aqueous solution on a CaF_2_ plate and gently dried under atmospheric pressure. In this case, the virtual absence of H_2_O background absorption allows the spectral range to be extended down to ∼130 nm. The spectrum of a dried myoglobin film is presented in Fig. 6 ▶, averaged over several rotation angles. Its line shape in the far-UV range resembles the myoglobin spectrum in aqueous solution (*cf*. Fig. 3*a* ▶), indicating that the protein is still in a predominantly folded conformation. There is an additional band between 130 and 160 nm, and it has been recently shown that the intensity and peak position of this band is sensitive to the secondary structure of a protein and may reflect changes in super-secondary and tertiary structure (Nesgaard *et al.*, 2008 ▶). These observations may pave the way towards discriminating between different types of super-secondary structures, especially for fibrillar and aggregated proteins and peptide species, by exploiting this information from the VUV spectral range.

## 6. Discussion and conclusions   

In the first three years of regular user operation, UV-CD12 has proven to be a valuable tool for structural biology research and lived up to the expected performance. Special precaution was taken to balance the photon flux compared with the former conditions at the SRS Daresbury to guarantee an excellent signal-to-noise ratio and short measurement time, while avoiding protein denaturation due to the intense UV light. Like all the other 9 existing SRCD beamlines worldwide, UV-CD12 has a standard experimental setup for static liquid-state experiments on proteins/peptides/nucleic acids, which is the basic equipment for all structural biology related work. In addition, based on the specialized expertise at IBG-2 and long-term solid-state NMR experience in the structural characterization of membrane-active peptides and transmembrane proteins in macroscopically oriented lipid samples (Fanghänel *et al.*, 2014 ▶; Strandberg *et al.*, 2013 ▶; Walther *et al.*, 2010 ▶), a new in-house-built SR-OCD experimental station has been integrated. It is the only SR-OCD based setup worldwide for solid samples with automated rotational averaging and data acquisition, and it provides improved spectral data quality especially for unsaturated and long-chain lipid environments at wavelengths <200 nm. This setup also includes humidity and temperature control accessories, allowing the influence of these critical conditions on the lipid phase state and hence the function of embedded bio-molecules to be studied. Due to the newly developed user-friendly control and data acquisition software with straightforward Macro programming, users have a maximum of flexibility when individually designing automated scans. In collaboration with the IR group at ANKA, who have experience with rapid-mixing microfluidic systems for time-resolved spectroscopy, there are plans to equip UV-CVD12 in the future with a new end-station for simultaneous SRCD and FTIR absorption measurements using a bench-top FTIR instrument. Such an extension would allow monitoring the kinetics of protein folding and other structural changes, thus exploiting the strong complementarity of the two techniques in the conformational characterization of bio-molecules.

## Supplementary Material

A 3-D view of the UV-CD12 beamline, spectral data that characterize the beamline performance (wavelength calibration, spectral resolution, stray light, CD scale calibration) and the `Material and Methods' data for the highlighted research results. Moreover, additional figures of control experiments for Fig. 3(c) and Fig. 5(a) and a table showing baseline noise levels and a table with worldwide operational SRCD beamlines.. DOI: 10.1107/S1600577515004476/ig5018sup1.pdf

## Figures and Tables

**Table 1:** Details of the beamline and experimental station

Beamline name	UV-CD12
Source type	Bending magnet (1.5T)
Photon beam divergence	30.8 mrad (H) 3.7mrad (V)
Mirror	Planar water-cooled mirror, silicon substrate coated with aluminium†
Monochromator	Toroidal holographic grating (300 lines mm^1^), peak output at 200nm, Al-coated† silica (HORIBA Yobin Yvon, Longjumeau, France)‡
Grating major radius (tangential)	10.42 0.02m
Grating minor radius (sagittal)	5.302 0.01m
Energy range	3.69.5eV
Wavelength range	130340nm
Beam size (at sample position)	2.5mm (H) 4.8mm (V) (at 200nm, 1nm spectral bandwidth)
Beam divergence at focus	7.4 mrad (H), 2.8mrad (V)
Photon flux§	1 10^12^ photons s^1^ (at 200nm, 1nm spectral bandwidth, 160mA electron beam current)
Spectral bandwidth	0.52.0nm (depending on variable 110mm exit slit width)
Typical acquisition parameters	Scan speed: 17nmmin^1^; lock-in time constant: 0.3s; dwell time: 1.5s
Experimental station	Rochon polarizer: MgF_2_, PUM 2.12 (B. Halle Nachfolger, Berlin)
	Photoelastic modulator: CaF_2_, PEM-90, 50kHz (Hinds Instruments, Hillsboro, USA)
	Sample mounting (see text and Fig. 2 ▶) (*a*) SR-LCD module: in-house-built U-shaped copper stage for liquid-state samples with temperature-controlled Peltier heat pumps for thermal ramping experiments (275368K). (*b*) SR-OCD module: in-house-built rotatable OCD cell for anisotropic solid samples (peptides in hydrated lipid membrane) with humidity and temperature control
Detector type	Photomultiplier tube (PMT)
Detector model	Electron tubes 9406B, or solar blind 9402B

† Before re-installing the original primary mirror and grating at UV-CD12 the quality of their aluminium coatings was checked by Horiba-Jobin-Yvon Company (manufacturer of the grating) and no deterioration was found. Therefore, the cleaned primary mirror and the grating were re-installed at UV-CD12 without applying any additional MgF_2_ protective coating.

‡ Detailed specifications of the grating have been given by Clarke Jones (2004 ▶).

§ The photon flux was measured using a calibrated PMT tube (ET 9406B) as the detector with all optical components installed.
