# Supplementary material for: UV-CD12: synchrotron radiation circular dichroism beamline at ANKA
Source: J Synchrotron Radiat. 2015 Apr 11;22(Pt 3):844–52. doi: 10.1107/S1600577515004476 (PMC4416691; doi:10.1107/S1600577515004476)
Supplement: Supplementary file 1 [file s-22-00844-sup1.pdf]

JOURNAL OF SYNCHROTRON RADIATION

Volume 22 (2015)

Supporting information for article:

UV-CD12: Synchrotron radiation circular dichroism beamline at ANKA

Jochen Bürck, Siegmar Roth, Dirk Windisch, Parvesh Wadhwani, David Moss and Anne S. Ulrich

S1. Material and Methods

S1.1. Wavelength calibration

Wavelength calibration was performed by measuring the absorbance of a reference solution of 4% holmium oxide in 10% perchloric acid, which was prepared according to (Weidner et al., 1986). The holmium peak positions were used to set up the calibration function for wavelength vs. grating stepping motor positions. The obtained holmium absorption curve is shown in Fig. S2A. Over the wavelength range scanned, all the holmium absorbance maxima were within 0.1-0.2 nm of the respective literature values. For wavelengths below 170 nm the calibration was done analogously using the peak maxima of the nitrogen purging gas absorption spectrum. A calibration spectrum is shown in Fig. S2B. Here, the deviation between calibrated and reference value was < 0.5 nm.

S1.2. Spectral resolution

The spectral resolution was determined using a 0.02% toluene solution in hexane as a reference (Starna GmbH, Pfungstadt, Germany) at 25°C. The absorption spectra for different exit slit widths are shown in Fig. S3. The spectral band width was determined from the peak ratio of the peak maximum absorbance closest to 268.7 nm and the minimum absorbance closest 267.0 nm, using tabulated values in the corresponding reference material certificate. These data confirmed an achievable spectral resolution range of 0.5 to 2.0 nm, meeting the design specification.

S1.3. Stray light

Stray light levels were determined by recording the transmission of calibrated aqueous solutions of sodium iodide (1%), lithium carbonate (saturated), and potassium chloride (1.2%) (Starna GmbH, Pfungstadt, Germany). The spectral scans are shown in Fig. S4. Transmission cut-offs for these materials are 260.8, 227.2 and 200.9 nm, respectively. The polychromatic stray light level was between 0.1 and 0.2% in the spectral range from 180-240 nm, which is within specification of the UV-CD12 setup.

S1.4. CD scale calibration

CD scale calibration of the beamline experimental setup was done using an aqueous solution of (1S)-(+)-camphor-10-sulfonic acid (CSA, ammonium salt) with a concentration of 9.72 mg/mL and a cuvette with an optical path length of 100 µm. NH_4CSA is commonly used as an ellipticity calibration standard for CD instruments, because it has a pronounced negative band at 192.5 nm and a positive maximum at 290.5 nm, with a ratio of the two bands of -2.0 (Venyaminov & Yang, 1996). Fig. S5 shows the corresponding spectrum measured at UV-CD12, and the observed ratio of -2.04 is in good agreement with the literature value.

S1.5. Preparation and SRCD measurement of aqueous myoglobin and human serum albumin solution

Horse skeletal muscle myoglobin (SIGMA-Aldrich, Schnelldorf, Germany) was dissolved in water at a concentration of 14.6 mg ml^{-1}, and loaded into a 2 µm-path length CaF_2 demountable cell (124.036, Hellma, Müllheim, Germany). Three scans were recorded over a wavelength range from 260 to 165 nm, with the same measurement parameters and the same data post-processing as for the $(KIGAKI)_3$ experiments (see below). For converting the spectral data into molar circular dichroism units (Δε), the exact concentration of the protein solution was determined based on the UV absorption at 280 nm. A molar extinction coefficient of 13980 L mol^{-1} cm^{-1} was used for myoglobin (Pace et al., 1995), which contains two Trp and two Tyr residues in its amino acid sequence.

SRCD measurements of human serum albumin (HSA) were performed to proof the absence of UV-induced denaturation (see Fig. 3C). HSA (SIGMA-Aldrich, Schnelldorf, Germany) was dissolved in water at a concentration of 3.33 mg ml^{-1} and measured in a CaF_2 demountable cell with 12.1 µm optical path length. Twenty consecutive single scans were recorded in direct succession over a wavelength range from 260-175 nm at maximum beam current of 160 mA and using the same measurement parameters and data post-processing as for the myoglobin and $(KIGAKI)_3$ measurements (cf. section S1.7). In another series of measurements a second HSA sample of the same concentration was measured with the same measurement parameters, but in this case the sample was exposed to the SRCD beam at 170 nm for 10 min. before each consecutive scan (see Fig. S6), which are harsh conditions never applied in a typical user experiment.

S1.6. Sample preparation of $(KIGAKI)_3$ in lipid vesicles

The $(KIGAKI)_3$ peptides were synthesized using standard Fmoc protocols and purified as described earlier (Wadhwani et al., 2012). The lipids 1,2-dimyristoyl-*sn*-glycero-3-phosphocholine (DMPC) and 1,2-dimyristoyl-*sn*-glycero-3-phospho-(1'-*rac*-glycerol) (DMPG) were purchased from Avanti Polar Lipids (Alabaster, AL, USA). The lipids were co-dissolved in chloroform/methanol at a molar ratio of 3:1 and small unilamellar vesicles (SUV) were prepared as described earlier (Wadhwani et al., 2012). For preparation of the final CD samples, aliquots of the peptide stock solutions in water were added to the liposome dispersion. The final peptide concentration in the $(KIGAKI)_3$ vesicle dispersions was around 135 µM, and the lipid concentration was 6.75 mM, resulting in a peptide-to-lipid (P/L) ratio of 1:50.

S1.7. Thermal ramping SRCD experiments of $(KIGAKI)_3$

UV-CD12 was used to measure SRCD spectra of the $(KIGAKI)_3$ peptide and a mutant in which Ile-8 was replaced by the non-natural fluorinated *D*-amino acid CF_3-Bpg (3-[trifluoromethyl]-*D*-bicyclopent-[1.1.1]-1-ylglycine), in a DMPC/DMPG 3:1 lipid dispersion in water. A 0.1 mm fixed path length

cylindrical quartz glass cell (Hellma, Müllheim, Germany) was used for the thermal scan SRCD experiments, with the aim of minimizing the background absorption of these samples resulting from H_2O and scattering artifacts caused by the lipid vesicles, which lead to increased noise levels in CD spectra measured at a bench-top instrument especially at wavelengths < 200 nm. Demountable cells with an even thinner optical path could not be used for these experiments, because the sample would dry out at higher temperatures due to the evaporation of water. With the help of the cylindrical cell it was possible to measure both peptides in lipid vesicles reliably down to 180 nm even at elevated temperatures. For the SRCD thermal scans the samples were thermostated at temperatures ranging from 30-80°C in 10° steps, and back using a 1 K min^{-1} heating/cooling rate, and a 5 min delay time at each temperature equilibration step between successive measurements. The temperature of the SR-LCD module described in section 2.2 of the paper and the thermal ramping process were automatically controlled by using an in-house data-acquisition software developed under the LabView environment. Under the SRCD conditions it was sufficient to collect only two scans from 260-180 nm at 0.5 nm intervals, using a fixed 1 nm spectral bandwidth, a 0.3 s lock-in time, a 1.5 s dwell time, and a scan-rate of 17 nm min^{-1}. SRCD spectra were processed with CDTool software, i.e. the averaged baseline of a peptide-free lipid reference sample measured at 30°C was subtracted from the averaged sample spectra of the thermal run, and finally the data were smoothened by a Savitzky-Golay algorithm contained in this software package (Lees at al., 2004).

S1.8. Cloning, expression and purification of PDGFRβ-TM domain, sample preparation of oriented lipid bilayers and SR-OCD measurements

The 39 amino acid stretch of the PDGFRβ transmembrane domain was produced recombinantly at the IBG-2. Cloning, expression and purification have been described in detail previously (Muhle-Goll et al., 2012). For OCD, the PDGFRβ transmembrane segment had to be reconstituted in oriented lipid bilayers. First, the lyophilized protein and 1,2-dieicosenoyl-*sn*-glycero-3-phosphocholine (DEiPC, Avanti Polar Lipids, Alabaster, AL, USA) lipid powder were co-solubilized in chloroform/methanol 1:1, and insoluble parts were removed by centrifugation. An aliquot of the protein/lipid solution was deposited onto a quartz glass plate, and the solution was allowed to dry in air. The amount of peptide and lipid on the plate was 1.58 and 79 nanomoles, respectively, with a P/L ratio of 1:50. For the second sample with a P/L of 1:500 the amount of peptide and lipid on the plate was 0.6 and 300 nanomoles. The samples then were placed under vacuum to remove residual organic solvents, followed by subsequent rehydration at 97% relative humidity overnight in the OCD cell shown in Fig. 2B, to obtain macroscopically aligned oriented lipid bilayers. OCD measurements on the J-810 desktop spectropolarimeter were performed as described earlier (Bürck et al., 2008, Muhle-Goll et al., 2012) in the wavelength range from 260-180 nm, using a 20 nm min^{-1} scan rate, an automated slit with nominal 1 nm spectral bandwidth, and a 4 s response time. At each of the 8 rotation angles one scan was acquired, and afterwards the average was

taken, and the rotationally averaged reference spectrum of the pure lipid was subtracted to obtain the final spectrum.

SR-OCD measurements of the same sample and in the same OCD cell at UV-CD12 were performed in the spectral range from 260-180 nm, using a scan-rate of 20 nm min^{-1} and comparable acquisition parameters, i.e. a lock-in time of 0.3 s, and a dwell time of 1.5 s, using a fixed spectral band width of 1 nm. For all OCD measurements the temperature of the sample was set to 30°C in order to stay above the lipid phase transition temperature.

For the control experiments on the J-810 instrument at different exit slit widths (see Fig. S7) a PDGFRβ/DEiPC sample with a P/L ratio of 1:50 was measured at each setting of the slit width (0.8, 1.4, 1.8, 2.2, 2.6, and 3.0 mm) using the same conditions as described above. However, no lipid baseline reference was measured and subtracted as these measurements were just intended to show the reduced signal magnitude of the OCD spectrum at increasing slit width.

For another control experiment with the J-810 due to lack of PDGFRβ peptide OCD spectra of the helical antimicrobial peptide PGLa in DErPC lipid bilayers (1,2-dierucanoyl-*sn*-glycero-3-phosphocholine, Avanti Polar Lipids, Alabaster, AL, USA) were measured at a P/L of 1:100. Here, a black cardboard with a punched out pinhole of 2.5 x 4.8 mm which mimicked the UV-CD12 beam geometry was centered in front of the OCD cell and spectra with and without pinhole were measured using the same measurement parameters as above. The corresponding OCD spectra are shown in Fig. S8.

S1.9. Dried myoglobin film sample

The measurement with the dried myoglobin film was performed using a flat CaF_2 plate as a support, which absorbs only below 130 nm. To measure the dried spectrum, the CaF_2 plate was fitted into the OCD cell holder at ambient humidity to maintain a stable orientation in the incident light beam. The plate was carefully cleaned with milliQ water and dried before a baseline spectrum was collected. Subsequently, 6 µL of a 1.265 mg mL^{-1} horse myoglobin solution in H_2O (0.4 nMol) were deposited on the CaF_2 plate and gently dried in air. Afterwards, the sample was dried under low vacuum (about 15 mbar) for 5 min and visually inspected for particles or opaque areas. The sample was transferred to the SR-OCD setup, and a sample spectrum was obtained in the wavelength range from 280-170 nm using the standard acquisition parameters shown in Table 1. A second series of measurement was done from 175-130 nm, using a lock-in time of 1 s, a dwell time of 5 s, and a scan rate of 5 nm min^{-1}. For obtaining a good signal-to-noise ratio at this low wavelength range, a total of 16 scans (2 at each of the 8 rotation angles) were averaged. Finally, the averaged spectrum of the empty CaF_2 plate was subtracted, and the two spectra from 280-170 and 175-130 nm were concatenated to give the final spectrum.

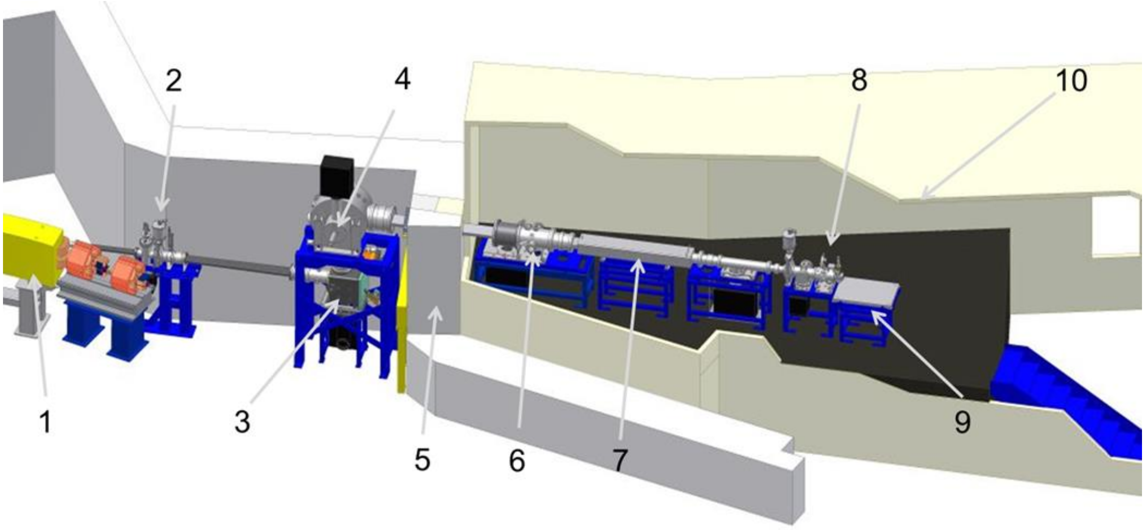

Figure S1 3D view of UV-CD12 at ANKA. The design model shows the main beamline components: (1) 1.5 T bending magnet source; (2) front-end with beam absorber, gate valve and fast closing shutter; (3) primary mirror chamber with water-cooled plane mirror; (4) monochromator chamber with toroidal grating; (5) radiation shield wall; (6) post-mono manual baffles; (7) acoustic delay section; (8) exit slit section with exit window; (9) optical table with SR-LCD/SR-OCD experimental setup; (10) experimental hutch.

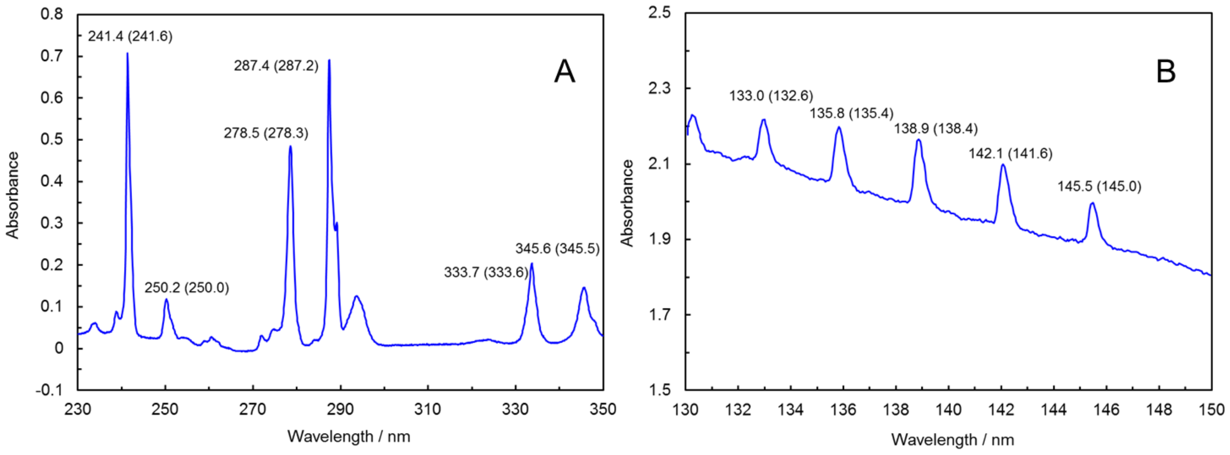

Figure S2 (A) Wavelength calibration using the absorption spectrum of 4% (m/v) holmium oxide in 10% $HClO_4$ solution, data in brackets are reference values from (Weidner et al., 1986); (B) Wavelength calibration below 160 nm with the absorption spectrum of dry nitrogen used for purging the sample chamber, data in brackets are reference values from (Trickle et al.,1995).

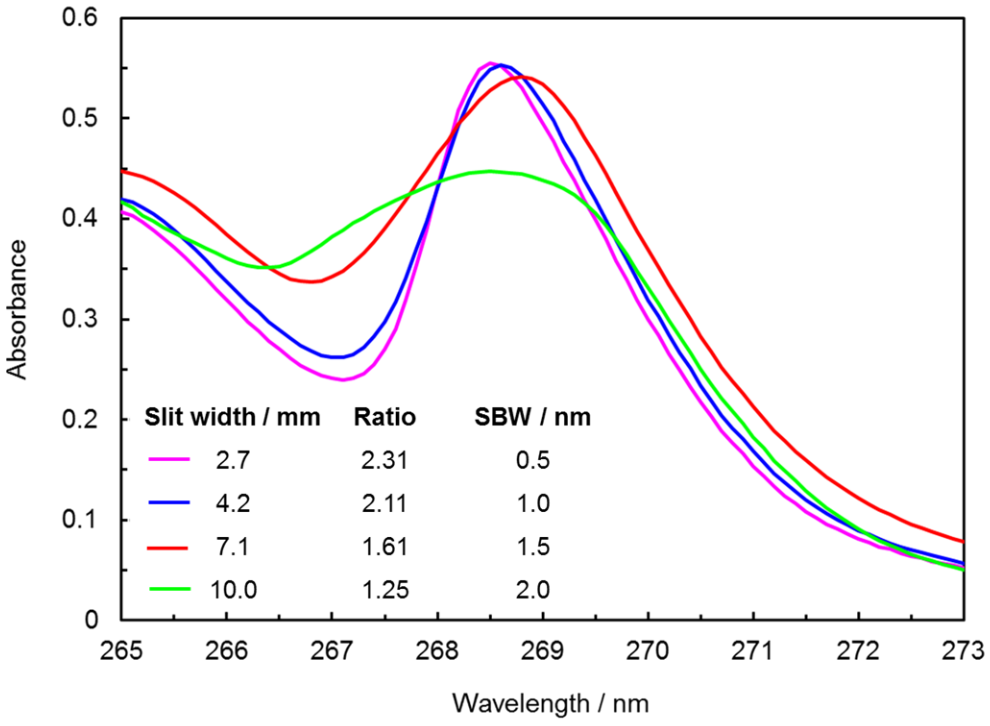

Figure S3 A 0.02% solution of toluene in hexane was measured at different exit slit widths. The peak ratio of the maximum to the minimum absorbance is a measure of the spectral band width, which is given in the table (inset).

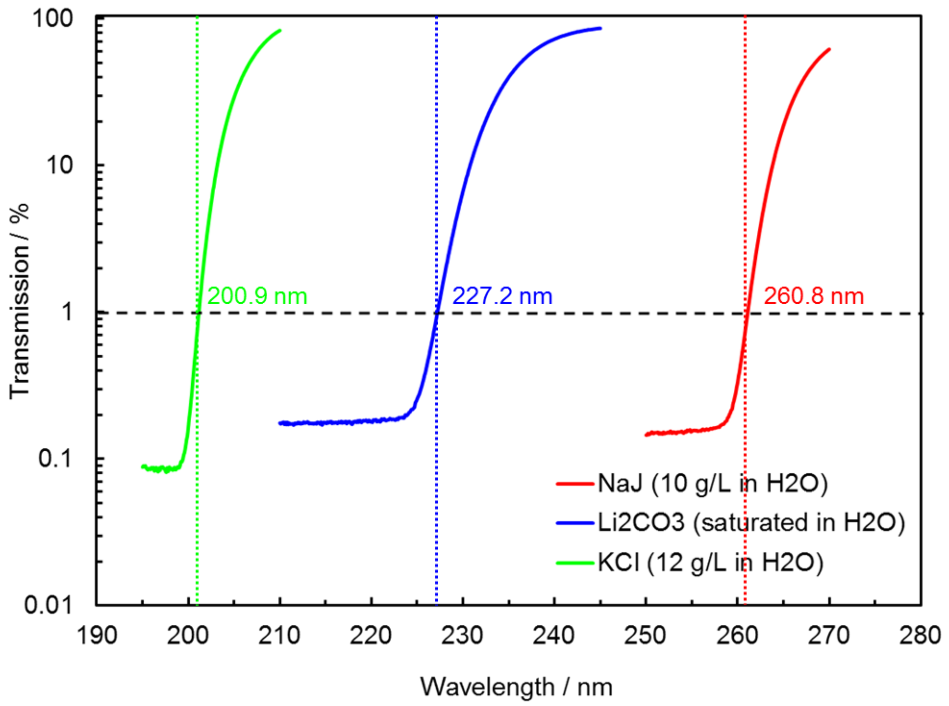

Figure S4 Transmission spectra of standard salt solutions were recorded on UV-CD12 to measure the level of polychromatic stray light, which is typically in a range between 0.1 and 0.2%.

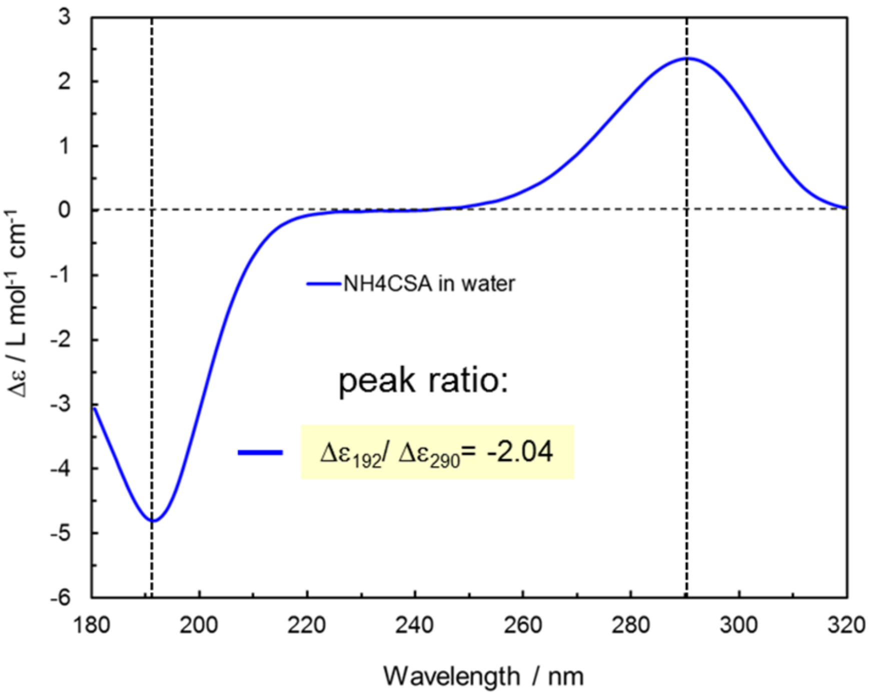

Figure S5 CD scale calibration of UV-CD12 using an aqueous solution of (1S)-(+)-camphor-10-sulfonic acid (ammonium salt), 9.72 mg/mL in H_2O, optical path length: 100 µm. The ratio of the two band maxima at 192.5 and 290.5 nm of -2.04 is in good agreement with the literature value.

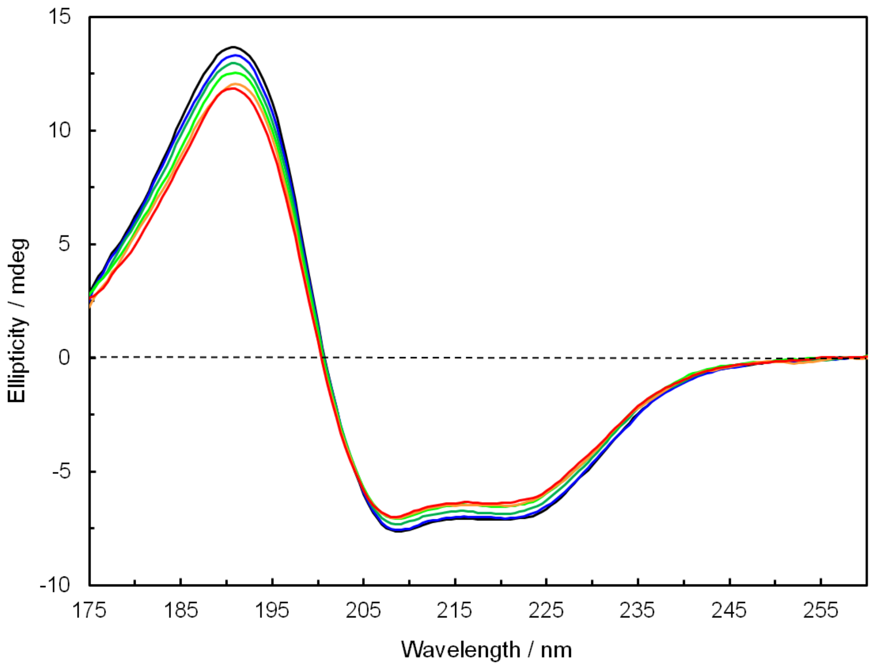

Figure S6 6 consecutive scans of human serum albumin collected at the UV-CD12 beamline with exposure of the sample to the SRCD beam at 170 nm for 10 min. after each scan (electron beam current was close to the maximum level of 160 mA). The black line indicates the first scan and the red line the last scan. Only for these “harsh” conditions with continuous irradiation of the sample with light of high energy and for an extended time of 10 min. between scans, which never would be applied in a typical user experiment, a gradual decrease of the signal magnitude is observed after each scan, which proofs the occurrence of UV-induced denaturation effects. However, for the normal user experiment conditions shown in Fig. 3C absolutely no degradation can be induced even after 20 scans.

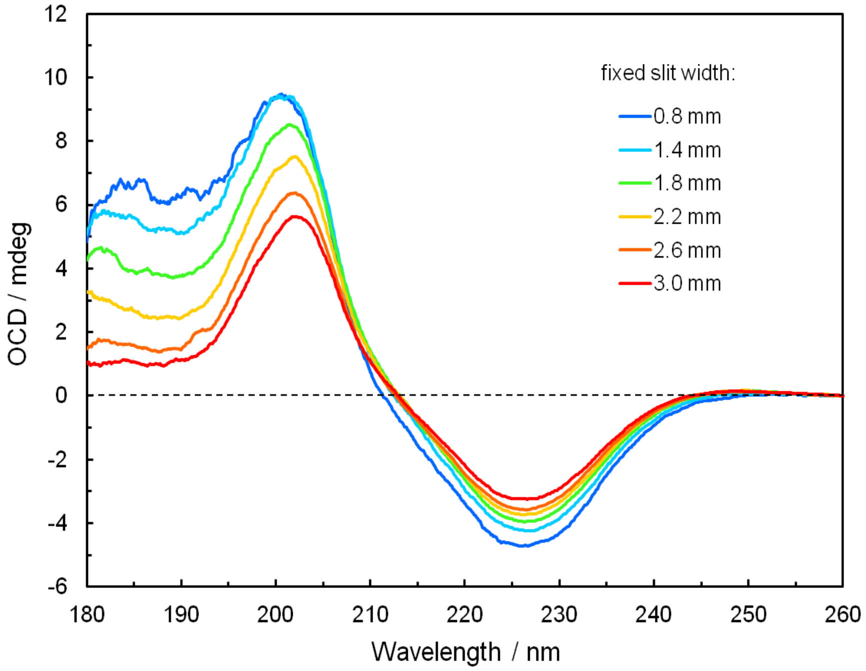

Figure S7 Comparison of OCD spectra of the transmembrane segment of PDGFRβ in fully hydrated oriented DEiPC lipid bilayers (P/L 1:50) measured on the J-810 and using varying fixed slit widths (here no lipid reference was subtracted). The measurements clearly demonstrate the loss in CD signal magnitude and spectral distortions when the exit slit is opened up.

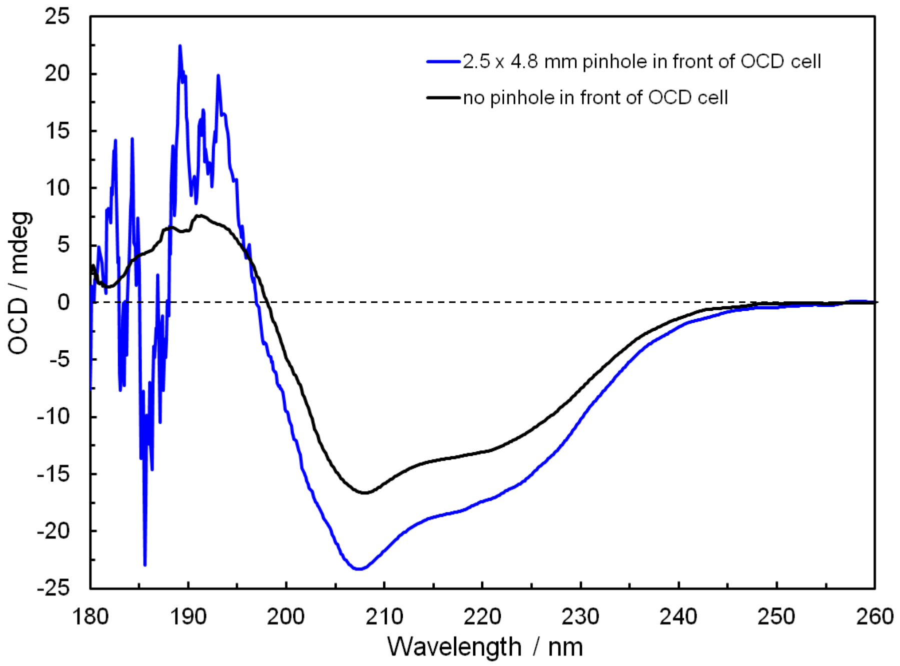

Figure S8 Comparison of OCD spectra of the α-helical antimicrobial peptide PGLa in fully hydrated oriented DErPC lipid bilayers (P/L 1:100) measured on the J-810 with and without pinhole in front of the OCD cell (the pinhole mimicks the UV-CD12 beam size). Here no lipid reference was subtracted. The signal magnitude is enhanced by ~40% when the pinhole is inserted, which demonstrates that the 4.3-fold bigger beam size of the J-810 hits also inhomogeneous "empty" zones at the outer rim of the sample which leads on average to a signal reduction compared with the UV-CD12 beam size geometry. For the J-810 measurement an increased noise level is obtained at wavelengths < 200 nm with the pinhole inserted because less photons reach the detector due to the reduced beam size.

Table S1 Root-mean squared (RMS) baseline noise level for SRCD measurements at UV-CD12; baseline noise was measured without sample cell under N_2 purging (flow rate of 20 L min^{-1}).

	RMS noise / mdeg*		
Wavelength / nm	Lock-in amplifier time constant: 3 s	Lock-in amplifier time constant: 1 s	Lock-in amplifier time constant: 300 ms
170	0.064	0.140	0.269
200	0.029	0.058	0.086
250	0.020	0.034	0.056
300	0.026	0.037	0.071

* Spectral band width: 1 nm; measured at maximum electron beam current of ~160 mA, RMS values have been determined from 60 consecutive measurements at each wavelength.

Table S2 Worldwide SRCD beamlines in operation (in alphabetic order, status February 2015)

Synchrotron	Beamline(s)	Location	Website
ANKA	UV-CD12	Germany	http://www.anka.kit.edu/1225.php
ASTRID2	AU-UV<br>AU-CD	Denmark<br>Denmark	http://www.isa.au.dk/facilities/astrid2/beamlines/AU-uv/AU-uv.asp<br>http://www.isa.au.dk/facilities/astrid2/beamlines/AU-cd/AU-cd.asp
BESSYII	UVIS	Germany	https://www.helmholtz-berlin.de/pubbin/igama_output?modus=einzel&sprache=en&gid=1692&typoid=37587
BSRF	4B8	China	http://english.ihep.cas.cn/rs/fs/srl/facilityinformation/beamlineintroduction/4B8/201203/t20120329_83222.html
DIAMOND	B23 (Module A/B)	U.K.	http://www.diamond.ac.uk/Beamlines/Soft-Condensed-Matter/B23.html
HiSOR	BL15	Japan	http://www.hsrc.hiroshima-u.ac.jp/english/bl15.htm
NSRRC	04B1	Taiwan	http://140.110.203.42/EFD.php?num=239
SOLEIL	DISCO	France	http://www.synchrotron-soleil.fr/Recherche/LignesLumiere/DISCO

New SRCD beamlines are in the planning phase at ALBA (Spain) and LNLS (Brazil).

S2. Supplementary references

Bürck, J., Roth, S., Wadhwani, P., Afonin, S., Strandberg, E. & Ulrich, A. S. (2008). *Biophys. J.* **95**, 3872–3881.

Lees, J. G., Smith, B. R., Wien, F., Miles, A. J. & Wallace, B. A. (2004). *Anal. Biochem.* **332**, 285–289.

Muhle-Goll, C., Hoffmann, S., Afonin, S., Grage, S.L., Polyansky, A. A., Windisch, D., Zeitler, M., Bürck, J. & Ulrich, A. S. (2012). *J. Biol. Chem.* **287**, 26178-26186.

Pace, C. N., Vajdos, F., Fee, L., Grimsley, G. & Gray, T. (1995). *Protein Sci.* **4,** 2411-2423.

Trickle, T., Proch, D. & Kompa, K. L. (1995). *Journal of Molecular Spectroscopy* **171**, 374-384.

Venyaminov, S. Y. & Yang, J. T. (1996). *Circular Dichroism and the Conformational Analysis of Biomolecules*, edited by G. Fasman, pp. 102-103. Plenum Press: New York/London.

Wadhwani, P., Strandberg, E., Heidenreich, N., Bürck, J., Fanghänel, S. & Ulrich, A. S. (2012). *J. Am. Chem. Soc.* **134**, 6512–6515.

Weidner, V. R, Mavrodineanu, R., Mielenz, K. D., Velapoldi, R. A., Eckerle, K. L. & Adams, B. (1986). SRM 2034, Holmium oxide solution wavelength standard from 240-640 nm, *NBS special publication* 260-102.
